# A novel functional electrical stimulation sleeve based on textile-embedded dry electrodes

**DOI:** 10.1186/s12938-024-01246-8

**Published:** 2024-06-04

**Authors:** Baptiste Garnier, Melissa Marquez-Chin, Stephanie DiNunzio, Stephanie N. Iwasa, Zia Saadatnia, Hani E. Naguib, Milos R. Popovic

**Affiliations:** 1 KITE-Toronto Rehabilitation Institute, University Health Network, Toronto, Canada; 2 Institute of Biomedical Engineering, University of Toronto, Toronto, Canada; 3 Department of Mechanical & Industrial Engineering, University of Toronto, Toronto, Canada; 4 Department of Mechanical and Manufacturing Engineering, Ontario Tech University, Oshawa, Canada

**Keywords:** Functional electrical stimulation, Dry electrodes, Sleeve, Smart textile, Rehabilitation

## Abstract

**Background:**

Functional electrical stimulation (FES) is a rehabilitation technique that enables functional improvements in patients with motor control impairments. This study presents an original design and prototyping method for a smart sleeve for FES applications. The article explains how to integrate a carbon-based dry electrode into a textile structure and ensure an electrical connection between the electrodes and the stimulator for effective delivery of the FES. It also describes the materials and the step-by-step manufacturing processes.

**Results:**

The carbon-based dry electrode is integrated into the textile substrate by a thermal compression molding process on an embroidered conductive matrix. This matrix is composed of textile silver-plated conductive yarns and is linked to the stimulator. Besides ensuring the electrical connection, the matrix improves the fixation between the textile substrate and the electrode. The stimulation intensity, the perceived comfort and the muscle torque generated by the smart FES sleeve were compared to hydrogel electrodes. The results show a better average comfort and a higher average stimulation intensity with the smart FES sleeve, while there were no significant differences for the muscle torque generated.

**Conclusions:**

The integration of the proposed dry electrodes into a textile is a viable solution. The wearable FES system does not negatively impact the electrodes’ performance, and tends to improve it. Additionally, the proposed prototyping method is applicable to an entire garment in order to target all muscles. Moreover, the process is feasible for industrial production and commercialization since all materials and processes used are already available on the market.

## Background

FES is a rehabilitation technique used to restore or improve motor control in paralyzed individuals [1–4]. It involves sending low-energy electric pulses through peripheral nerves of the affected limb to activate the concerned muscles [5]. This rehabilitation method is particularly effective for the rehabilitation of stroke and spinal cord injury patients [6, 7]. Despite the clinical successes, the way by which the stimulation is delivered is cumbersome, as it requires at least two hydrogel electrodes per stimulated muscles, which are linked to the stimulator using long cables [8, 9]. This is not a major issue for the stimulation of one or two muscles, but to achieve more complex tasks, such as reaching, grasping and walking, that involve 6 to 12 stimulation electrode pairs, the use of hydrogel electrodes and individual cables is prohibitively cumbersome. Consequently, more sophisticated forms of FES therapy must be delivered in a clinical setting, as they require a knowledgeable therapist to deliver the treatment. Thus, a new method for delivering FES therapies is needed. To address this problem, various groups started developing dry electrodes, with the objective of using them as reusable stimulation electrodes [10]. One of the benefits of dry electrodes is that they are reusable and can be used without the need for conductive gel. A wide range of materials can be used to create dry electrodes [11], making them easily adaptable for FES applications [12–14]. However, one of the fundamental problems with dry electrodes, as metal plate or carbon rubber, is that they are uncomfortable and individuals using them feel pain and discomfort during stimulation [15]. Indeed, due to their high conductivity, metal plate electrodes can cause severe skin burns and require to be associated with a wet medium to homogeneously distribute the current. Similarly, carbon rubber electrodes, which have a lower conductivity, require gel or water as skin interface, making them difficult to use for long-term applications. Recently, our group developed a novel dry electrode based on polyvinylidene fluoride (PVDF) thermoplastics and Carbon nanotube (CNT) conductive fillers for FES to address these particular challenges [16]. The gel electrode provides less comfort and more skin contact for accurate measurements [16]. The fluorine-based materials offer a significant advantage in terms of surface smoothness. At present, they are still permitted by regulations, but they are at risk of being banned in the near future, especially in the European Union, due to the REACH regulation [17].

To make the FES more convenient, different wearable devices have been proposed [18]. However, the use of textiles in the form of sleeves or leggings could provide faster placement of the electrodes and potentially better ergonomics. The textile-based device could also enable individuals with impaired hand function to don and doff the electrodes independently. Progress in printing technology has enabled the development of printed electrodes on a textile substrate [19]. However, the porous nature of textile materials makes it difficult to control the amount of ink and, thus the electrode properties [20]. On the other hand, knitting technology enables the development of garments, such as sleeves or tights, integrating textile conductive yarns to create textile electrodes in specific locations [21]. However, developed textile electrodes are not suitable for FES applications due their discomfort. Indeed, the textile electrodes can cause uncomfortable sensations, such as stinging, sharp, burning, stabbing, pricking, pulling, tingling, shooting and throbbing, depending on each individual. Despite better performances in terms of flexibility or stretchability, they also require, at least, to be wet before stimulation [22]. Moreover, conventional textile materials and processes produce rough electrode surfaces that do not match the skin’s roughness impedance [23]. The same applies to textile electrodes produced by embroidery technology, except that the electrodes are embroidered onto the textile substrate after its fabrication, in contrast to knitted electrodes, which are produced during the knitting process [24].

Consequently, this study aims to develop a new wearable device for FES that addresses the above discussed challenges, namely, (i) reusability, (ii) minimize pain and discomfort, (iii) make donning and doffing easier, and (iv) simplify the connection to the stimulator and electric pulses transfer. The innovative character of the proposal lies in the device’s manufacturing, the materials used, and the associated processes. The proposed prototype is composed of a knitted sleeve that embeds carbon-based dry electrodes and a textile conductive yarns system to connect it to the MyndSearch stimulator. The electrodes are melted and compressed onto a nonwoven thermoadhesive and a conductive matrix, made of textile silver-plated conductive yarns, sewn onto the textile substrate. The textile conductive yarns are then connected to the stimulator thanks to a crimp connector. Thus, the described prototyping method can be applied to produce a smart FES garment for a wide range of muscles. The wearable nature of the prototype makes it particularly suitable for long-term applications, such as FES therapy. This article describes the materials used, prototyping methods deployed, as well as functional results, including comfort evaluation and muscle torque generated using stimulation on the biceps.

## Results

### Stimulation comfort

**Fig. 1:**
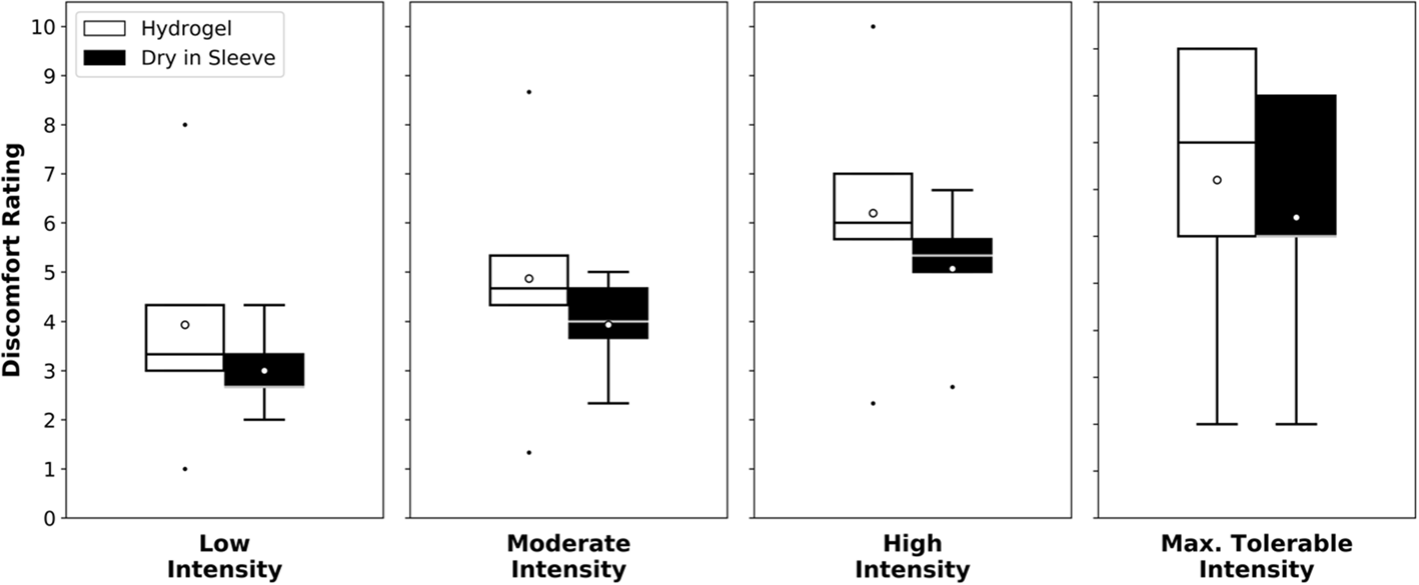
Average perceived comfort rating where 0 = most comfortable and 10 = most uncomfortable. In each boxplot, the horizontal line indicates the median and the circle the mean

The stimulation comfort represents an evaluation of the pain felt by the subject during the stimulation. The patient rates its comfort from 0 (most comfortable) to 10 (most uncomfortable). This measurement allows the establishment of if the device is suitable for functional electrical stimulation therapy. For reported comfort ratings, participants rated the sleeve with the dry electrodes to be, at least, as comfortable as the hydrogel electrodes on average for all intensity levels, as seen in Fig. 1. For the maximum tolerable intensity, the average discomfort was 6.40 ± 2.88 with the sleeve, and 7.20 ± 3.35 with the hydrogel electrodes. At high intensity, the average discomfort rating was 5.07 ± 1.48 with the sleeve and 6.20 ± 2.75 with the hydrogel electrodes. At moderate intensity, the average discomfort was 3.93 ± 1.04 with the sleeve and 4.87 ± 2.62 with the hydrogel electrodes. At low intensity, the average discomfort rating was 3.00 ± 0.88 with the sleeve, and 3.93 ± 2.58 with the hydrogel electrodes.

### Stimulation-induced muscle torque

**Fig. 2:**
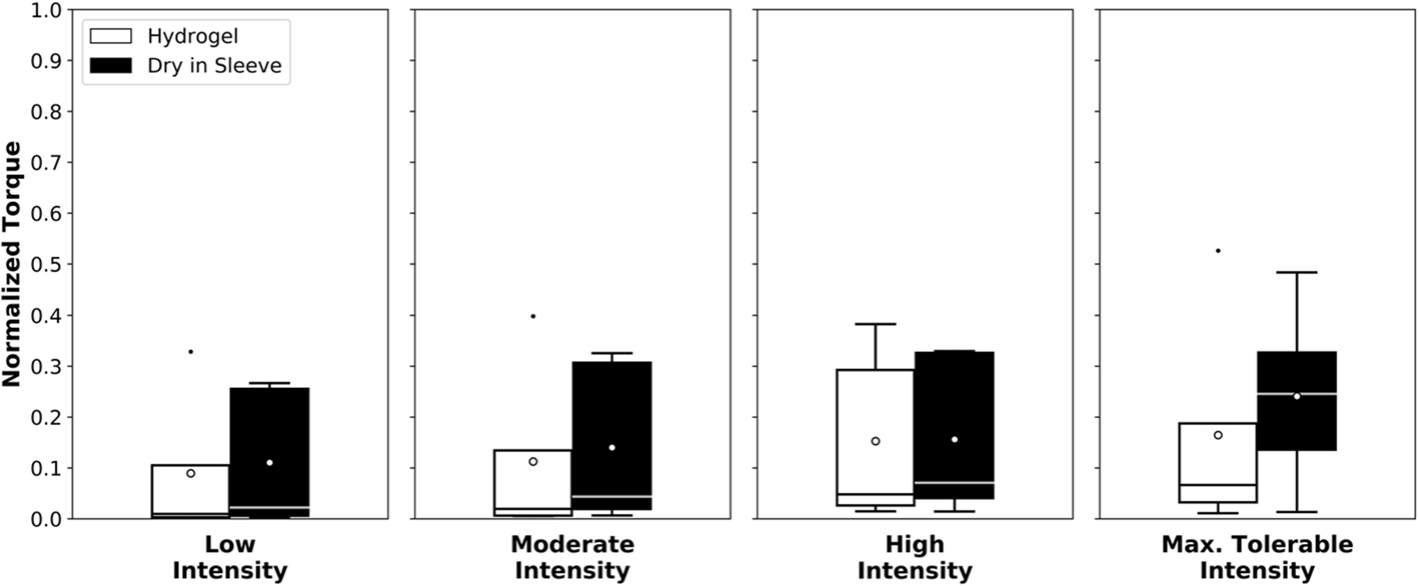
Average normalized torque induced with stimulation. In each boxplot, the horizontal line indicates the median and the circle the mean

The measurement of the stimulation-induced muscle torque allows evaluation of the stimulation’s effectiveness to induce movement. The muscle torques produced with stimulation on average were not significantly different with either electrode type at any intensity level and are shown in Fig. 2. At the maximum tolerable stimulation intensity, the average normalized torque produced was 0.241 ± 0.180 with the sleeve and 0.164 ± 0.213 with hydrogel electrodes. At high intensity, the average torque with the sleeve was 0.156 ± 0.158 and 0.153 ± 0.172 with the hydrogel electrodes. At moderate intensity, the average torque was 0.140 ± 0.161 with the sleeve and 0.113 ± 0.169 with the hydrogel electrodes. At low intensity, the average torque with the sleeve as 0.111 ± 0.138 and 0.089 ± 0.141 with the hydrogel electrodes.

### Stimulation intensities used

**Fig. 3:**
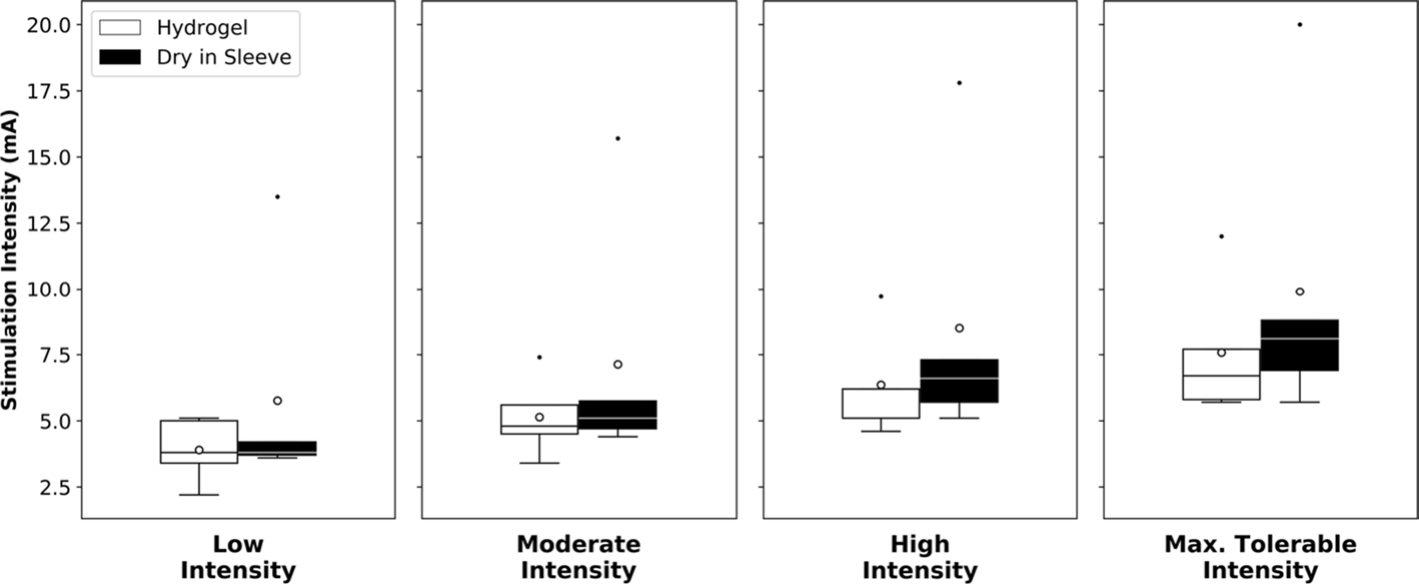
Average stimulation intensity values used across all participants. In each boxplot, the horizontal line indicates the median and the circle the mean

The stimulation intensity is given by the stimulator, and allows comparison of the electrical behavior between the different electrodes. The stimulation intensities used for all five participants, seen in Fig. 3, were higher on average with the dry electrodes in the sleeve than with the hydrogel electrodes. At the maximum tolerable level, the average stimulation used was 9.90 ± 5.77 mA with the sleeve, and 7.58 ± 2.60 mA with the hydrogel electrodes. For the high level, the average intensity was 8.50 ± 5.27 mA with the sleeve and 6.36 ± 1.99 mA with the hydrogel electrodes. For moderate levels, the average intensity was 7.13 ± 4.82 mA with the sleeve and 5.14 ± 1.49 mA with the hydrogel electrodes. For low levels, the average intensity was 5.76 ± 4.33 mA with the sleeve and 3.90 ± 1.20 with the hydrogel electrodes.

### Stimulation sensations reported

**Fig. 4:**
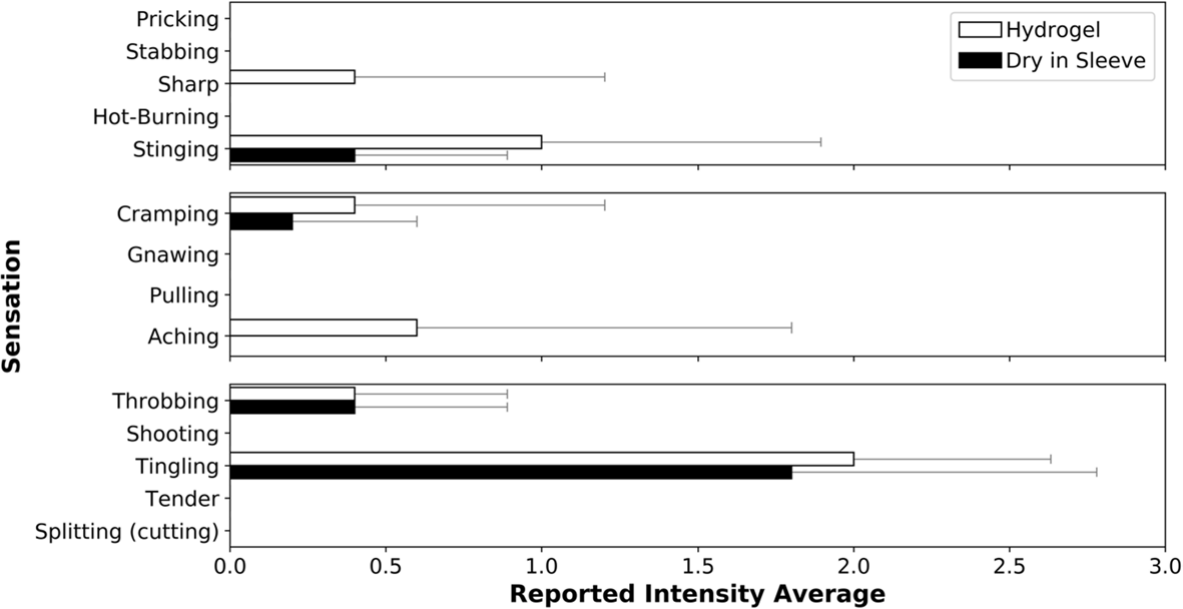
Average reported sensations after stimulation with each electrode type

The average reported sensations after stimulation are shown in Fig. 4. The reported stimulation sensations provide descriptions of the sensations for each electrode. The most reported sensation on average was ‘tingling’ for both electrode types, with individuals rating the intensity on average 1.80 ± 1.10 for the sleeve and 2.00 ± 0.71 for the hydrogel electrodes. Other reported sensations were ‘stinging’, ‘throbbing’, and ‘cramping’ with the sleeve and ‘stinging’, ‘aching’, ‘sharp’, and ‘cramping’, and ‘throbbing’ with the hydrogel electrodes. The sensation intensities were rated on average at 0.40 ± 0.55 with the sleeve and 1.00 ± 1.00 with the hydrogel electrodes for ‘stinging’, 0.40 ± 0.55 with both sleeve and hydrogel electrodes for ‘throbbing’, 0.20 ± 0.45 with the sleeve and 0.40 ± 0.89 with the hydrogel electrodes for ‘cramping’, and 0.60 ± 1.34 for ‘aching’ and 0.40 ± 0.89 for ‘sharp’ with the hydrogel electrodes.

## Discussion

The results presented above represent a significant advancement in the FES rehabilitation of patients who have experienced strokes or spinal cord injuries. Indeed, this study demonstrates the feasibility of a flexible and wearable FES system that presents similar performances, in terms of intensity level, comfort and torque, as hydrogel electrodes. Thus, the method of integrating the dry electrodes into a wearable textile structure and the flexible connections system do not impact their performances. This would, in the long run, facilitate a broader adoption of this treatment by simplifying its use for healthcare professionals and making it more comfortable for patients. However, dry electrodes, by definition, are not covered in sticky gel, which can lead to electrode shifts during muscle contraction or limb movement. Furthermore, the spacing between electrodes is fixed, which may not necessarily suit all body types, despite the use of three different sizes. In this study, these measurements and spacing were appropriate for the 5 study participants. However, since the position of the electrodes is fundamental for comfort and effectiveness of the FES therapy, future wearable systems could need precise tailoring. Thus, the development of custom-made smart FES clothing, as is already practiced for compression stockings for example, could prove to be an adequate solution. The choice of the conductive matrix design may also be a subject of inquiry. In our case, sewing the conductive textile yarns in straight parallel lines has been chosen for its quick implementation and repeatability.

Then, a study focusing on mechanical and chemical robustness, as well as the reliability of the prototype, is underway to establish its reusability. Indeed, a challenge for this device is its resistance to washing. Given its current design and construction, there are several solutions available to protect the conductive parts from mechanical and chemical stresses experienced during washing cycles. For example, the use of covering embroideries or a polyurethane film to protect the conductive tracks are solutions being considered [25].

Furthermore, even though this study only presents a device for anterior biceps brachii flexion, this manufacturing method is applicable to almost all parts of the body. Only the pattern making, and the tailoring steps of the textile substrate need to be adapted to the chosen limb. This multidisciplinary approach to FES also opens new possibilities for this therapy, such as its integration with wireless communication [26] and/or brain-controlled neuromodulation [27].

Finally, a clinical study including more participants is planned to consolidate these results to obtain approval from healthcare services and thus enables widespread dissemination.

## Conclusions

This paper presents the manufacturing process of a smart FES sleeve. It describes the materials used and the prototyping steps implemented to manufacture the smart sleeve. The prototype is composed of a knitted sleeve made of polyester, viscose and elastane that integrates carbon-based dry electrodes and electrical tracks connected to the MyndSearch stimulator. The carbon-based dry electrodes are fixed on the textile substrate by thermal compression onto a nonwoven thermoadhesive (“light weight non-woven fusible interfacing’ from Pellon&Tailor^®^ Company) and a sewn conductive matrix made of textile silver-plated conductive yarns. The yarns composing the matrix are twisted and crimped into a standard circular connector, and then inserted into shrink tubing. The textile conductive matrix provides an electrical connection between the electrodes and the stimulator. The sleeve pattern was designed as a compression garment for the upper arm to ensure a good skin/electrode contact. The tests conducted with stimulation, showed that the smart FES sleeve performance, in terms of stimulation intensities, perceived comfort and muscle torque, are at least as good as the hydrogel electrodes. Thus, the proposed method of integrating dry electrodes into wearable textile structure is viable and allows for easier use of dry electrode for FES therapy. Additionally, the proposed design can also be implemented towards developing full FES garments, such as shirt, pants, and so on. Finally, since all the described processes and materials are already on the market and used by the industry, this manufacturing method is conceivable for industrial production.

## Materials and methods

### Dry electrode

The dry electrode used to develop the smart FES garment is based on our previous study [16]. Briefly, it is made of polyvinylidene fluoride (PVDF) mixed with 5% in weight of carbon nanotube (CNT). Then, the polymer is shaped into a thin, flexible, and conductive film thanks to a standard solution casting process, which can be easily tailored to desired sizes. Its initial degradation temperature is 383 ± 2 °C, its elastic modulus is 1.19 ± 0.04 GPa, and its yield strength is 16.3 ± 0.1 MPa. Regarding the electrode’s electrical behavior, its average impedance, from 0 to 1 MHz, is 401.19 ± 664.63 KΩ, and its average surface resistivity is 261.66 ± 85.42 Ω [16].

### Textile conductive yarn

A major novelty of the smart FES sleeve lies in the method of connecting the electrodes through textile materials. While sewing or embroidering conductive textile yarns is already widely used to create conductive tracks on textile substrates [28–30], the creation of a sewn conductive matrix as a contact power supply support for dry electrodes is unprecedented. The conductive matrix consists of 7 parallel stitch lines made with two “Silver-Tech HC12” yarns (sewing and bobbin), from Madeira Company [31]. This yarn presents a linear resistivity around 100 Ω.m^−1^ [32]. The use of conductive threads for both sewing and bobbin allows for an electrical connection between both sides of the textile, thus ensuring the connection of both the electrode inside the sleeve and the stimulation outside the sleeve.

### Textile substrate

A blend of polyester (80%), viscose (15%) and elastane (5%) has been used to develop smart FES sleeve. As a support, the textile sleeve must ensure accurate positioning and good adhesion of the electrodes, as well as withstand the electrode fixation process, which includes thermal compression molding at 190 °C. This structure has also been chosen because it provides elasticity and ensure a good skin–electrode interface.

### Textile sleeve pattern

**Fig. 5:**
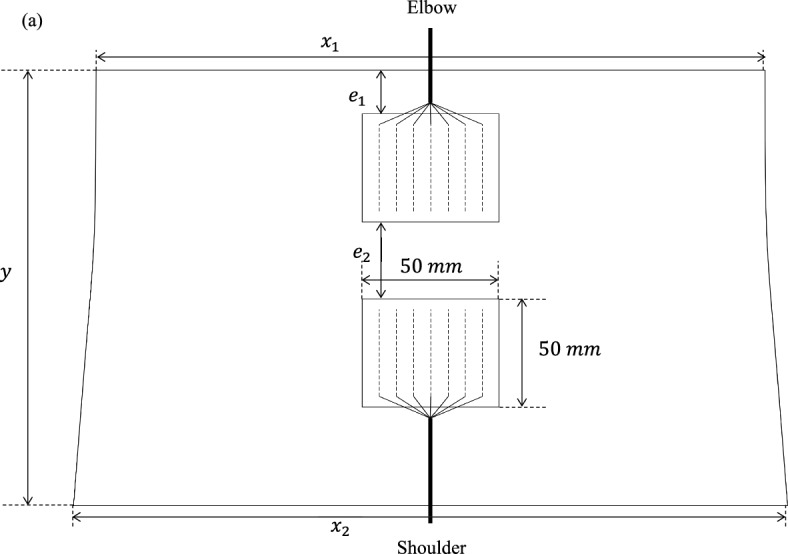
Textile pattern of the smart FES sleeve, with its specific dimensions

The knitted substrate was cut according to a specific pattern to shape a sleeve. The textile sleeve pattern is the 2-dimensional representation of the sleeve before it was assembled, as presented in Fig. 5. It is created based on the patient’s biceps measurement and the positions of the electrodes, with the aim of stimulating the anterior brachial biceps, responsible for elbow flexion. Three different sizes (S, M, L) have been developed, and their dimensions are displayed in Table 1. The Inkscape^®^ software was used to design the pattern.

**Table 1:** The corresponding size guide of the smart FES biceps sleeve

Size	${x}_{1}$	${x}_{2}$	$y$	${e}_{1}$	${e}_{2}$
S	21 cm	23 cm	19 cm	2 cm	3.5 cm
M	23.5 cm	25 cm	20.5 cm	2.3 cm	4.2 cm
L	26 cm	29 cm	21.5 cm	2.5 cm	5 cm

### Textile electrode conductive interface

**Fig. 6:**
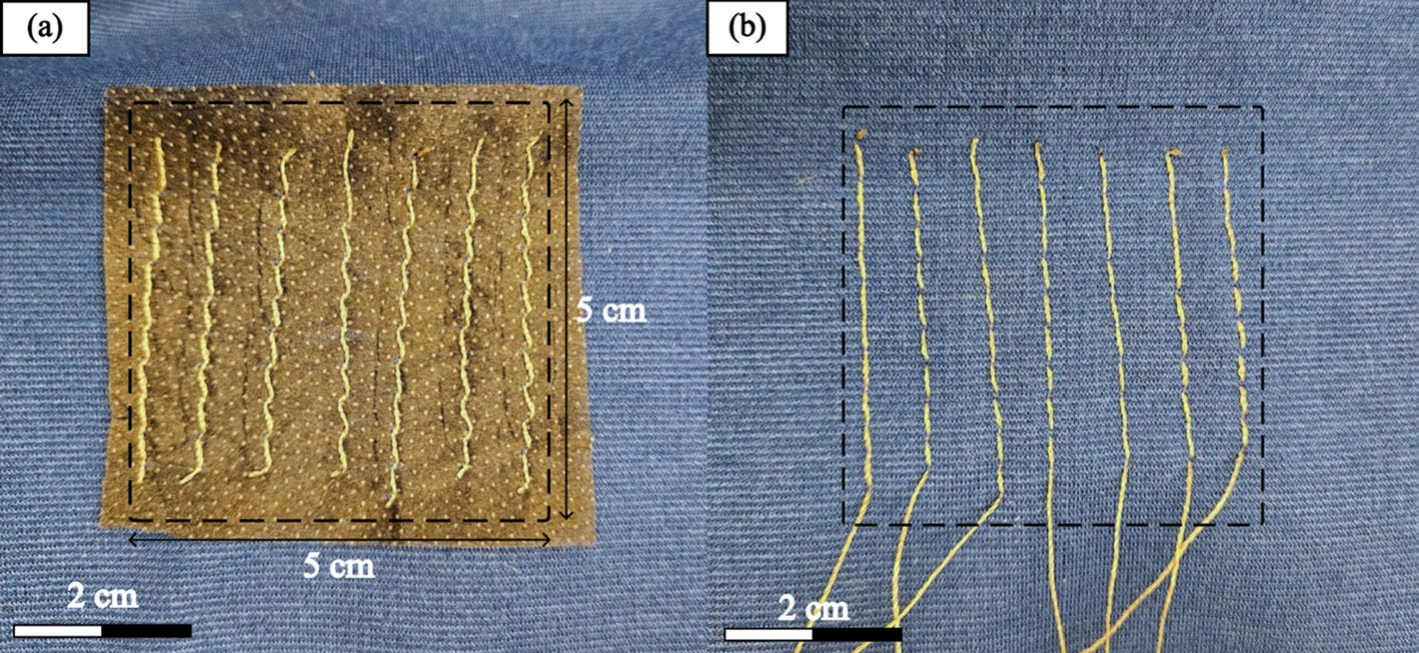
Sewn conductive matrix on the knitted substrate and the nonwoven thermoadhesive. **a** The inner side (facing skin) and **b** the outer side

The textile conductive yarns created the electrical connection between the dry electrode and the stimulator. They form a conductive matrix, fixed by sewing or embroidery, on the knitted substrate. Figure 6 presents a sample of the embroidered conductive matrix on the knitted substrate, both the front and the back sides. The textile conductive yarns composing the matrix were extended to a non-insulated quick-disconnect crimp connector with a diameter of 2 mm and a length of 2 cm, allowing them to carry the current from the stimulator to the electrode. Additionally, the use of sewn textile conductive yarns provides a larger contact surface, improving the mechanical and chemical fixation. Moreover, the matrix’s roughness enabled the electrode’s polymer to fill the new volume under thermal compression, enhancing mechanical fixation.

The matrix is composed of seven parallel textile conductive yarns. This geometry ensured a homogeneous current distribution inside the matrix, and consequently in the electrode.

### Dry electrode fixing

**Fig. 7:**
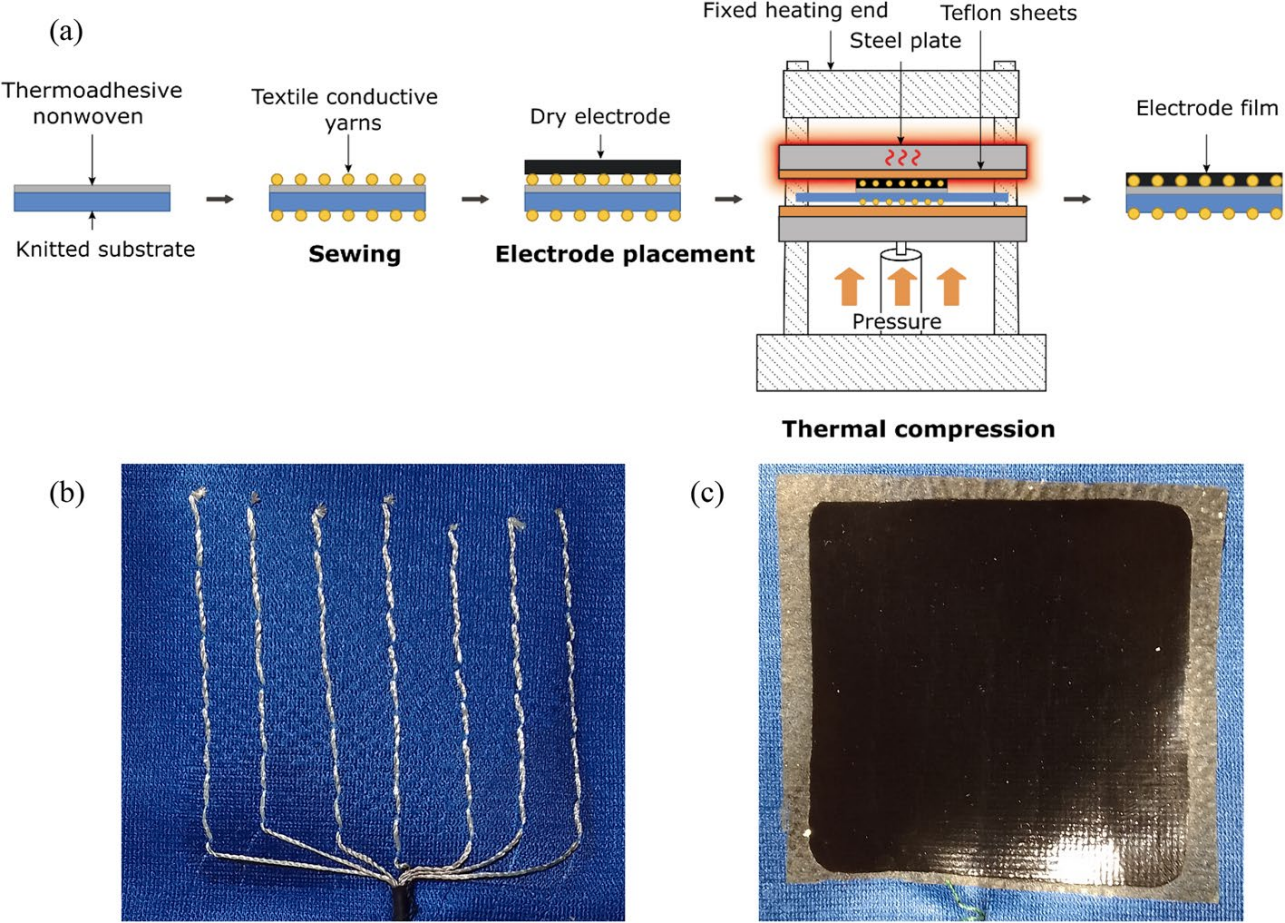
**a** Process of attaching the dry electrode to the textile substrate. Conductive textile threads are sewn onto the knitted and nonwoven thermoadhesive structure, then the dry electrode is fixed by thermal compression. Photo of the outer **b** and inner **c** side

Once the textile conductive matrix was formed, the carbon-based dry electrode was fixed on the substrate through a thermal compression process, as described in Fig. 7(a), using a CARVER Compression Molding Press. It enables the melting and compression of the dry electrode onto the textile substrate under conditions similar to those during its fabrication. The machine applied a temperature of 190 °C solely from the top side. From the bottom side, 1 ton pressure was applied for 5 min, followed by 5 tons pressure for 5 to 7 min. The area of the compression mold press was a 15 × 15 cm square shape. To increase the surface smoothness, two Teflon sheets were placed on the steel plate to sandwich the textile substrate and the electrode. After the thermal compression, the sample was removed once it cooled down to room temperature. Consequently, the CNT dry electrode is directly melted and compressed on the conductive matrix, making this thermal compression process the main element of the fixation. The thermoadhesive layer enables to aid the fixation, especially at the electrode’s corners. Photographs of both sides of the fixed dry electrode on the knitted substrate are presented in Fig. 7(b, c).

### Tailoring

**Fig. 8:**
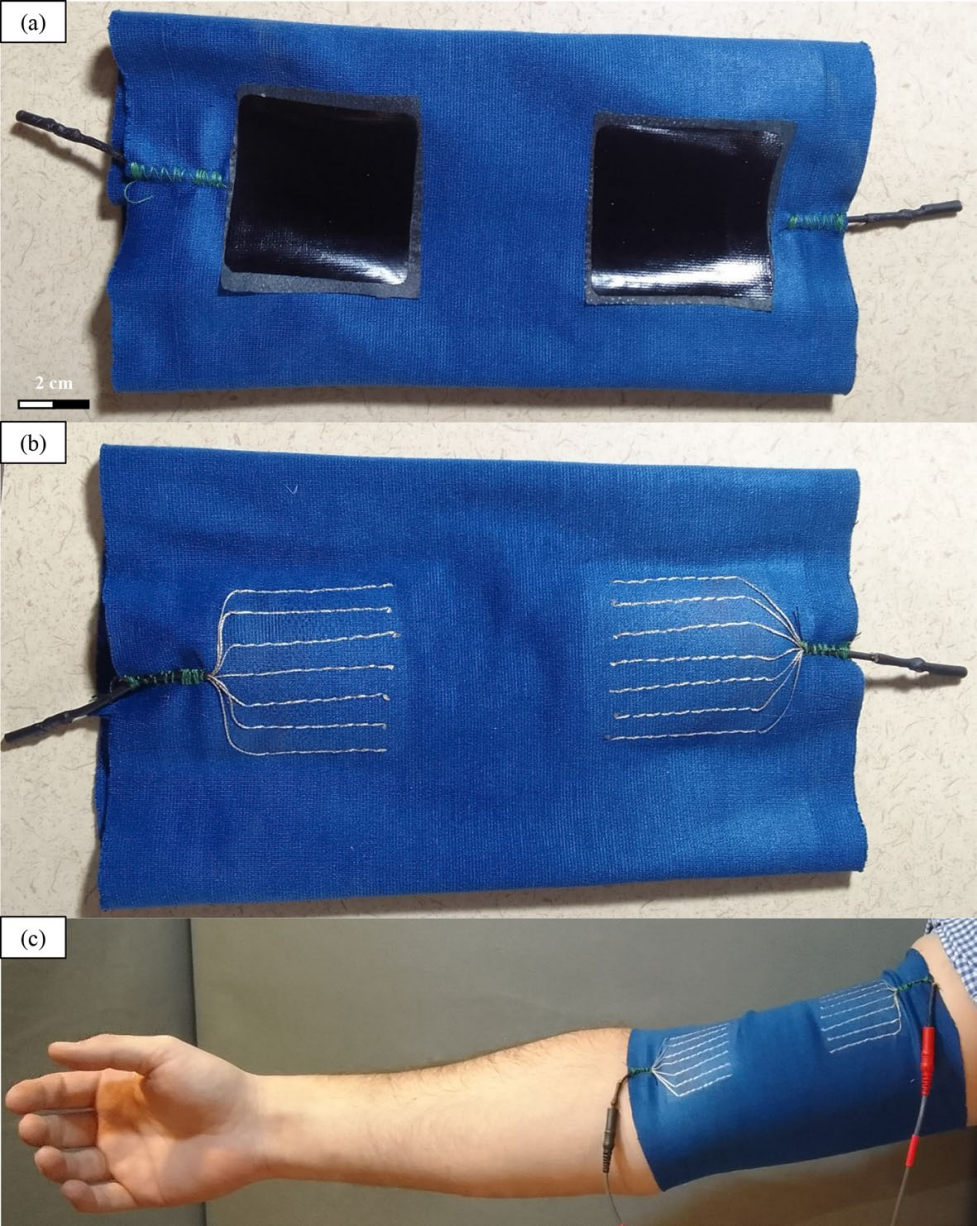
The smart FES sleeve for biceps: **a** the inner and **b** the outer sides, and **c** the prototype worn and connected

The last step of the manufacturing process consisted of tailoring the pattern to shape it into a sleeve and integrate the connectors. The remaining protruding textile conductive yarns from the conductive matrix are twisted and then inserted into a circular crimp connector, which is then threaded into shrink tubing. The resulting cables were fixed on the substrate using cording stitches. The textile structure (flat) is finally closed to form a sleeve with straight stitches. The prototype of the smart FES sleeve for biceps is presented in Fig. 8.

### Experimental protocol of the smart FES sleeve characterization

The purpose of the smart FES sleeve for the biceps was to provide an elbow flexion through electrical stimulation of the anterior biceps brachii without causing pain. The stimulation was delivered to the right arm using the MyndSearch stimulator (MyndTec Inc.), with a pulse frequency of 40 Hz, a pulse width of 300 µs, and a pulse amplitude dependent on each individual. The study focused on comparing the proposed smart FES sleeve, which embeds dry electrodes, against conventional self-adhesive hydrogel electrodes (ValuTrode 5 × 5 cm, Axelgaard Manufacturing Co.) in terms of muscle torque and perceived comfort. The electrical intensity, the perceived comfort and the muscle torque were evaluated for 3 levels of stimulation intensity, with both the hydrogel electrodes and the smart FES sleeve for five participants, following the protocol outlined by our group [16]. Torque was measured using a Biodex dynamometer (System 3, Biodex Medical Systems) and comfort was rated using a 1 to 10 visual analog scale displayed in front of participants as well as a sensations questionnaire. All participants were informed of the protocols and signed a consent form approved by University Health Network’s Research Ethics Board (ID#21–5298). The three levels of stimulation intensity used to perform the measurements were defined as follows [16]:

$$\left\{\begin{array}{c}{\text{Low}}={\text{mCT}}+0.25*\left({\text{MTC}}-{\text{mCT}}\right)\\ {\text{Moderate}}={\text{mCT}}+0.5*\left({\text{MTC}}-{\text{mCT}}\right)\\ {\text{High}}={\text{mCT}}+0.75*\left({\text{MTC}}-{\text{mCT}}\right),\end{array}\right.$$

where mCT is the minimum contraction threshold, and MTC the maximum tolerated contraction threshold.

### Participants demographics

Five participants (3 female and 2 male, mean age 28.6 ± 2.7 years old), are presented in Table 2.

**Table 2:** Participant demographics

Age (years)	Sex	Weight (kg)	Height (m)	Dominant hand
32	Male	80	1.73	Right
30	Female	67	1.65	Right
25	Female	65.8	1.70	Right
29	Female	66	1.63	Right
27	Male	72.6	1.78	Right

## Data Availability

The data sets used and/or analyzed during the current study are available from the corresponding author upon the reasonable request.
